# Metabolic Profiling-based Data-mining for an Effective Chemical Combination to Induce Apoptosis of Cancer Cells

**DOI:** 10.1038/srep09474

**Published:** 2015-03-31

**Authors:** Motofumi Kumazoe, Yoshinori Fujimura, Shiori Hidaka, Yoonhee Kim, Kanako Murayama, Mika Takai, Yuhui Huang, Shuya Yamashita, Motoki Murata, Daisuke Miura, Hiroyuki Wariishi, Mari Maeda-Yamamoto, Hirofumi Tachibana

**Affiliations:** 1 Division of Applied Biological Chemistry, Department of Bioscience and Biotechnology, Faculty of Agriculture, Kyushu University, 6-10-1 Hakozaki, Higashi-ku, Fukuoka 812-8581, Japan; 2 Innovation Center for Medical Redox Navigation, Kyushu University, 3-1-1 Maidashi, Higashi-ku, Fukuoka 812-8582, Japan; 3 National Food Research Institute, National Agriculture and Food Research Organization, 2-1-12 Kannondai, Tsukuba, Ibaraki 305-8642, Japan; 4 Food Functional Design Research Center, Kyushu University, 6-10-1 Hakozaki, Higashi-ku, Fukuoka 812-8581, Japan

## Abstract

Green tea extract (GTE) induces apoptosis of cancer cells without adversely affecting normal cells. Several clinical trials reported that GTE was well tolerated and had potential anti-cancer efficacy. Epigallocatechin-3-*O*-gallate (EGCG) is the primary compound responsible for the anti-cancer effect of GTE; however, the effect of EGCG alone is limited. To identify GTE compounds capable of potentiating EGCG bioactivity, we performed metabolic profiling of 43 green tea cultivar panels by liquid chromatography–mass spectrometry (LC–MS). Here, we revealed the polyphenol eriodictyol significantly potentiated apoptosis induction by EGCG *in vitro* and in a mouse tumour model by amplifying EGCG-induced activation of the 67-kDa laminin receptor (67LR)/protein kinase B/endothelial nitric oxide synthase/protein kinase C delta/acid sphingomyelinase signalling pathway. Our results show that metabolic profiling is an effective chemical-mining approach for identifying botanical drugs with therapeutic potential against multiple myeloma. Metabolic profiling-based data mining could be an efficient strategy for screening additional bioactive compounds and identifying effective chemical combinations.

*Tea* (*Camellia sinensis* L.) is one of the most widely consumed beverages in the world. Epidemiological studies have associated green tea intake to reduced risk of prostate cancer, leukaemia and non-Hodgkin lymphoma^1^, and several clinical studies have suggested that green tea extract (GTE) could be an effective therapy for premalignant lesions in high-risk subjects^2^,^3^. Furthermore, a phase II trial of GTE in patients with chronic lymphocytic leukaemia (CLL) showed that GTE has an anti-CLL effect^4^. Moreover, unlike many potential anti-cancer drugs, green tea polyphenol is well tolerated by patients, and GTE has been approved by the United State Food and Drug Administration as the first botanical drug^5^. A recent study demonstrated that the green tea polyphenol epigallocatechin-3-*O*-gallate (EGCG) induces apoptotic cell death of cancer cells without affecting normal cells^6^.

Multiple myeloma (MM) accounts for approximately 13% of all hematologic cancers^7^. It is characterized by the secretion of Bence Jones protein^8^. High-dose chemotherapy followed by stem cells transplantation is among the most effective current regimens for MM^9^. However, MM is still difficult to cure and requires long-term disease control. Recent studies reported that the 67-kDa laminin receptor (67LR) is the target molecule of EGCG^10^,^11^,^12^ overexpressed in MM cells^13^,^14^ and acts as a cancer-specific death receptor when bound by EGCG^15^,^16^,^17^. This finding suggested that 67LR could be a novel target for chemotherapy and indicates a potential mechanism for the anti-cancer efficacy of EGCG. However, EGCG has selective cytotoxic effects on MM cells only at concentrations greater than 20 μM, considerably greater than the concentrations reached in clinical trials^4^.

Mass spectrometry (MS) is used for metabolomic research on plants, and liquid chromatography–mass spectroscopy (LC–MS) can detect a wide range of low-molecular-weight compounds such as secondary metabolites^18^. Metabolic profiling can highlight the association between the metabolites and phenotype^19^. Coupled with chemometric methods, including principal component analysis (PCA) and orthogonal partial least-squares (OPLS) regression analysis, it is often used for evaluating nutritional value in plant cultivars and to identify compounds conferring beneficial properties^20^. It is possible that this approach may also be useful for the unbiased evaluation of the pharmaceutical properties of crude plant extracts and to identify specific bioactive compounds in extracts. However, metabolic profiling for evaluating the anti-cancer properties of GTE compounds has been little studied.

In this study, we show that LC–MS-based metabolic profiling can reveal an effective chemical combination of GTE-derived compounds with high apoptosis induction capacity against MM cells. A polyphenolic compound, eriodictyol, was identified as a potentiator of the *in vivo* apoptosis-inducing effect of EGCG by a multivariate statistical analysis method capable of evaluating differences in metabolic profiles and anti-cancer effects of diverse GTEs.

## Results

### Comparison of apoptosis induction by GTEs from individual cultivars on the human MM cell line U266

**Figure 1:**
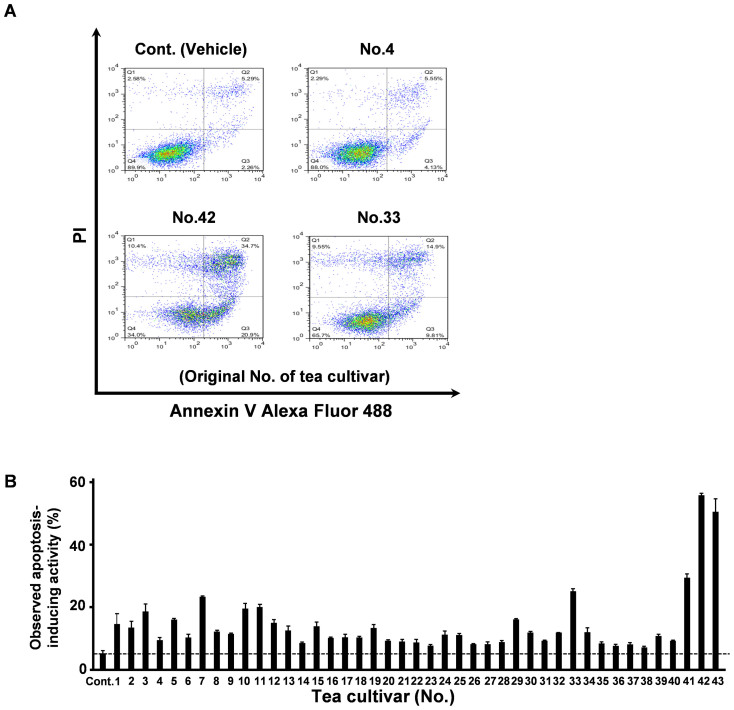
Apoptosis induction in human MM cells by 43 GTEs derived from separate cultivars. (A) U266 cells were treated with each green tea extract (GTE) for 96 h. Apoptotic cells were stained with Annexin V-Alexa Fluor 488 and propidium iodide and then apoptosis quantified using flow cytometry. All data expressed as mean ± SEM (*n* = 3). (B) Apoptosis-inducing activity (%) was calculated from the total population of Annexin V-positive cells.

Several clinical trials have shown the potential of GTE as an anti-cancer agent^2^,^3^,^4^,^21^. There are numerous green tea cultivars; however, most of which have not been tested for apoptosis induction of cancer cells. We investigated the apoptosis-inducing effects of GTEs from 43 green tea cultivars (Supplementary Table S1) on the human MM cell line U266 by annexin/PI double staining and flow cytometry. As shown in Fig. 1A–B, Supplementary Fig. S1 and Supplementary Table S1, the 43 cultivars showed variable potency for apoptotic induction. Some induced apoptosis in a substantial fraction of U266 cells after 96 h, particularly Nou-6, Sunrouge (SR) and Benifuki (BF), whereas others exhibited weak effects, such as the standard cultivar Yabukita (YB), a popular Japanese cultivar.

The chemical compositions of these extracts were then examined by LC–MS to identify those compounds with greatest apoptosis-inducing potency, either alone or in combination.

### Metabolic profiling-based data-mining for screening of effective GTE-derived chemical combinations for induction of apoptosis

**Figure 2:**
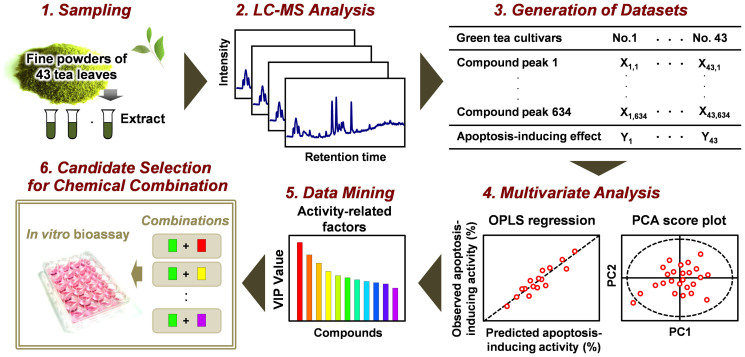
Experimental design of bioactive compound screening using metabolic profiling of 43 GTE panels from different cultivars. The green tea leaves were drawn by Motofumi Kumazoe.

**Figure 3:**
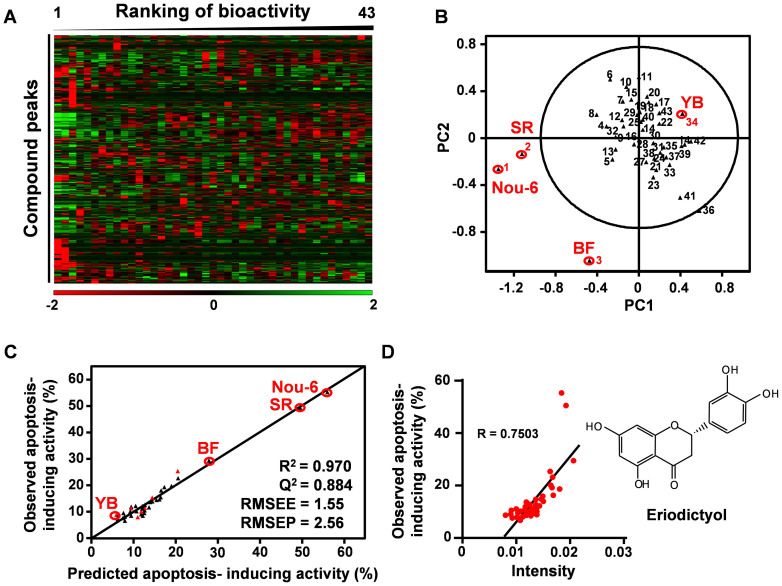
Metabolic profiles of GTEs for identifying sensitizers of EGCG pro-apoptotic activity. (A) Heat map analysis shows different component patterns among the 43 GTE panels. Columns represent the metabolic profile of single cultivars, rows represent 634 compound peaks. (B) PCA score plot shows different clusters of MS profiles, corresponding to highly bioactive Nou-6, Sunrouge and Benifuki, and those with low bioactivity, including the standard cultivar Yabukita. (C) Bioactivity-prediction OPLS model was calculated from LC–MS dataset of 36 tea samples as the training set and 43 tea samples included in the training set and the test set (red symbol). (D) Correlation plots between amounts of eriodictyol in the 43 GTEs and their apoptosis-inducing activity.

The chemical compositions of the extracts derived from 43 green tea cultivars were measured by LC–MS and subjected to multivariate statistical analysis to identify strong apoptosis induces (Fig. 2). Metabolic profiles differed markedly among green tea cultivars (Fig. 3A). Among PCA clusters, one comprised several cultivars with high apoptosis-inducing potency (Nou-6, BF, and SR), and another comprised the remaining cultivars, including YB (Fig. 3B). These results strongly suggest that chemical differences among cultivar extracts account for observed differences in bioactivity. We accordingly performed further analyses to identify the composition of each GTE and the individual compounds responsible for apoptosis induction.

The predominant phytochemical in GTE, EGCG, has clearly demonstrated apoptosis-inducing activity; however, the potential of other compounds to act synergistically with EGCG has not been examined. To identify candidate compounds potentiating the anti-cancer effect of EGCG from bioactivity-related composition profiles (Fig. 3A–B), we created an OPLS regression model using GTE composition profiles and bioactivity (Fig. 3C). The quality of the regression model indicated good predictive reliability, as verified in part by the values of the goodness-of-fit parameter R^2^ (0.970), the goodness of prediction parameter Q^2^ (0.884), the root mean squared error of the estimation (RMSEE, 1.55) and the root mean squared error of prediction (RMSEP, 2.66), indicated good predictive reliability of the model. This result suggests that the apoptosis-inducing effects of the 43 GTEs were explained by their composition profiles. In this model, compounds explaining predicted apoptosis-inducing effects were also identified by variable importance in projection (VIP) values. Large VIP values (>1) correspond to best explanations of predicted bioactivity. To screen candidates for effective anti-apoptotic combinations, 15 compound peaks with high VIP ranking were selected, and eight peaks (corresponding to EC, theanin, ECG, EGCG, theobromine, eriodictyol, Cya-glu and Cya-gal) were assigned (Supplementary Fig. S2 and Supplementary Fig. S3A). Among these compounds, only eriodictyol significantly potentiated the anti-cancer effect of EGCG (Supplementary Fig. S3B). A positive correlation was observed between the amount of eriodictyol in each GTE (LC–MS signal intensity) and the apoptosis-inducing potency against U266 cells *in vitro* (Fig. 3D). Surprisingly, naringenin and hesperetin, two analogues of eriodictyol, also significantly potentiated the anti-cancer effect of EGCG (Supplementary Fig. S4A–B). These findings suggest the utility of metabolic profiling for identifying effective anti-cancer drug combinations from raw GTEs.

### Eriodictyol potentiates apoptosis induction by EGCG in MM cells and the anti-tumour effect of EGCG in a mouse MM tumour model

**Figure 4:**
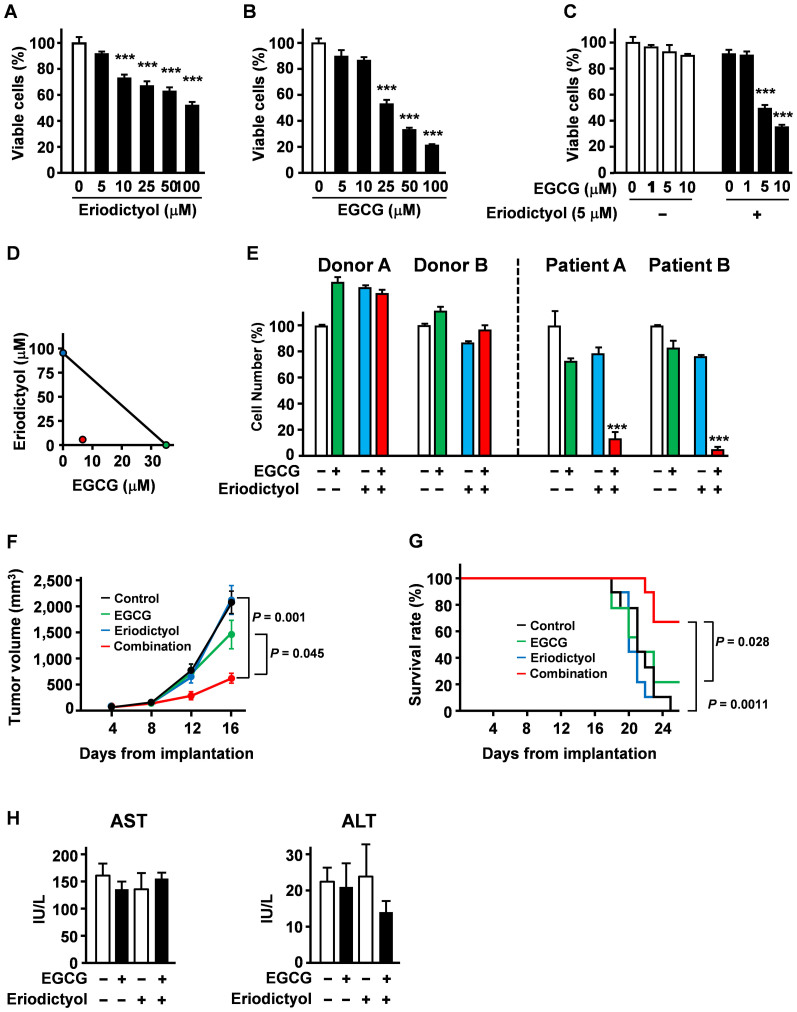
Eriodictyol significantly potentiates the anti-MM efficacy of EGCG *in vitro* and in mice. U266 cells were treated with (A) eriodictyol or (B) EGCG for 96 h and viable cell numbers measured by the ATPlite OneStep assay. (C) U266 cells were cultured with or without eriodictyol (5 μM) and/or indicated concentrations of EGCG for 96 h, and viable cell numbers measured. (D) Isobologram analysis revealed the synergism of the eriodictyol plus EGCG combination. (E) Normal peripheral blood mononucleated cells from two healthy donors and primary multiple myeloma cells from two patients were treated with eriodictyol (5 μM) and EGCG (5 μM) for 96 h. (F, G) MPC-11 cells were injected subcutaneously into mice (*n* = 9 per group). The mice were then injected with EGCG (15 mg/kg i.p.) and/or eriodictyol (15 mg/kg) every 2 days. (H) Serum levels of transaminases were evaluated (*n* = 7–9). All data are mean ± SEM.

To determine the effect of eriodictyol on the anti-cancer effects of EGCG, we performed isobologram analysis, a well-established technique to evaluate synergism based on the IC_50_ values of each drug and their combination^22^. The IC_50_ of eriodictyol was 97.7 μM (Fig. 4A) and that of EGCG was 35.3 μM (Fig. 4B). Pretreatment with only 5 μM eriodictyol significantly potentiated apoptosis of U266 cells by EGCG, reducing the IC_50_ from 35.3 μM to 6.6 μM (Fig. 4C). Isobologram analysis of growth-inhibition curves revealed that the combination of EGCG and eriodictyol was greater than additive (Fig. 4D). In contrast, eriodictyol alone exhibited no effect on the number of viable peripheral blood mononucleated cells (PBMCs) from healthy human donors, but significantly enhanced the cytotoxicity of EGCG on primary MM cells from patients (Fig. 4E).

To determine the therapeutic efficacy of combined EGCG and eriodictyol on tumour growth *in vivo*, mice inoculated with MPC-11 cells were treated with eriodictyol and EGCG. The combination slowed tumour growth significantly compared with saline or either compound alone (Fig. 4F), and log-rank analyses showed a significant increase in the survival time of mice treated with this combination compared with mice treated with saline, EGCG alone or eriodictyol alone (Fig. 4G). This combination did not increase serum levels of ALT/AST (Fig. 4H), indicating no substantial hepatotoxicity.

### Eriodictyol synergistically potentiates the anti-tumour effect of EGCG by amplifying 67LR-dependent signalling in MM cells

**Figure 5:**
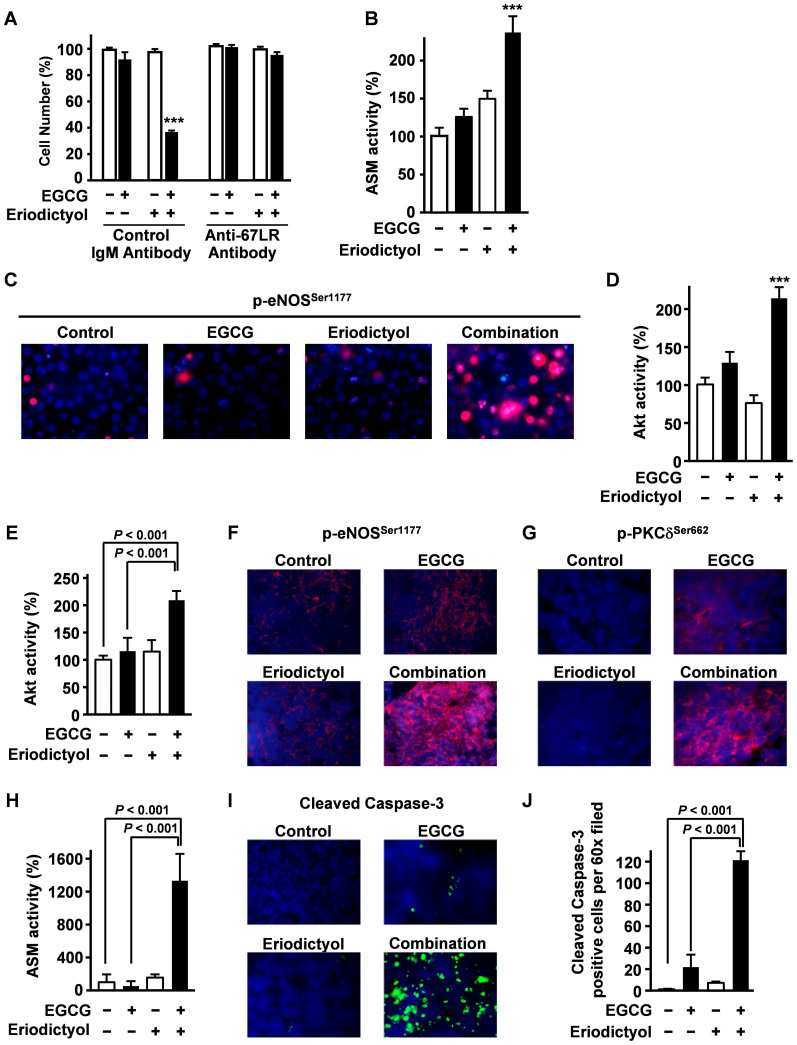
Eriodictyol synergistically potentiates the apoptosis-inducing activity of EGCG by enhancing the 67LR−ASM signalling pathway. (A) U266 cells were pretreated with anti-67LR antibodies or control IgM antibodies for 3 h, then treated with EGCG (5 μM) and eriodictyol (5 μM) for 96 h. (B) To determine the effect of eriodictyol (5 μM) and EGCG (5 μM) on ASM activity, U266 cells were treated with eriodictyol (5 μM) and/or EGCG (5 μM) for 3 h. (C, D) The effect of 1 h treatment with eriodictyol (5 μM) and EGCG (5 μM) in combination on (C) phosphorylation level of eNOS at Ser1177 and (D) Akt activity. Effect of EGCG and eriodictyol in combination on myeloma cell proliferation *in vitro*. (E–J) MPC-11 cells were injected subcutaneously into female BALB/c mice, and mice (*n* = 5 per group) were given single i.p. injections of EGCG (15 mg/kg i.p.) and/or eriodictyol (15 mg/kg i.p.). After 6 h, tumours were excised and evaluated for (E) Akt activity, (F) phosphorylation of eNOS at Ser1177, (G) phosphorylation of PKCδ at Ser662 (corresponding to human p-PKCδ Ser664) and (H) ASM activity. Representative images shown at ×60 magnification. (I, J) Cleavage of caspase-3 was evaluated by immunofluorescence analyses. Original magnification, ×60. All data are expressed as mean ± SEM.

EGCG is a known 67LR ligand, and 67LR binding appears to mediate the cytotoxic effect against cancer cells^11^,^12^,^13^,^14^,^15^. To determine if the EGCG/eriodictyol combination also induces 67LR-dependent cell death, we measured combined toxicity against U266 cells in the presence of a 67LR antibody. Consistent with 67LR-mediated cell death, the antibody blocked apoptosis reduction in response to both drugs alone and their combination (Fig. 5A). We previously reported that EGCG elicited 67LR-dependent cell death in MM cells through acid sphingomyelinase (ASM) activation^14^. The combination of EGCG and eriodictyol significantly induced ASM activation in U266 cells (Fig. 5B). It is known that EGCG induces ASM activation through Akt-mediated phospho-activation of endothelial nitric oxide synthase (eNOS) at Ser1177^16^,^17^, and the present study showed that eriodictyol significantly potentiated EGCG-induced Akt activation and eNOS phosphorylation at Ser1177 (Fig. 5C–D), indicating that potentiation of EGCG apoptosis induction by eriodictyol is mediate by enhanced activation of Akt/eNOS/ASM signalling.

To determine if this signalling pathway also mediates the synergistic anti-MM effects of EGCG plus eriodictyol *in vivo*, mice with palpable tumours following MPC-11 cell inoculation were injected with 15 mg/kg EGCG (i.p.), 15 mg/kg eriodictyol (i.p.), or both, and signalling activity examined in tumour tissue lysates by colorimetric enzyme assays (Akt, ASA) and tissue sections by fluorescence immunohistochemistry (protein kinase C delta (PKCδ)and eNOS. Injection of EGCG plus eriodictyol significantly increased Akt activity (Fig. 5E), enhanced phosphorylation of eNOS at Ser1177 (Fig. 5F), phosphorylation of PKCδ at Ser662 (corresponding to human PKCδ Ser664) (Fig. 5G) and ASM activity (Fig. 5H). Furthermore, eriodictyol dramatically potentiated EGCG-induced cleavage of the apoptosis effector caspase-3, a crucial triggering event for receptor-dependent apoptosis (Fig. 5I, J). Taken together, these results indicate that eriodictyol potentiated the anti-MM effect of EGCG by amplifying the 67LR/Akt/PKCδ/ASM signalling pathway.

## Discussion

We report the first application of metabolomics to identify potential anti-cancer compounds in crude extracts from multiple cultivars of green tea. We succeeded in identifying eriodictyol, a polyphenol compound that potentiated the apoptosis-inducing potency of EGCG against MM cells by about six-fold. Moreover, eriodictyol proved non-toxic against human PBMCs but did enhance EGCG-induced apoptosis of human MM cells derived from patients. In addition, this chemical combination reduced tumour growth rate and enhanced the survival of mice inoculated with MM cells to a significantly greater extent than EGCG alone. These new findings suggest the application of metabolic profiling techniques for evaluating the pharmacological effects of compounds in raw plant extracts and screening for anti-cancer compounds or synergetic sensitizers. This metabolomic screening approach with supervised multivariate OPLS regression analysis could be a valuable strategy for preclinical identification of anti-cancer compounds.

Although EGCG, the most active component of GTE, has selective toxicity against cancer cells that overexpress 67LR, the overall anti-tumour effect is limited. Indeed, the IC_50_ of EGCG is about 20–30 μM, much higher than the plasma concentrations of 5–7 μM achieved in clinical studies^21^. Our data show that eriodictyol can sensitize U266 multiple myeloma cells to plasma concentration of EGCG (IC_50_ of 6.6 μM in the presence of eriodictyol) by enhancing the 67LR-mediated Akt/eNOS/PKCδ/ASM signalling pathway without deleterious effects on normal cells. Hepatotoxicity is the main adverse effect of EGCG^23^. In some clinical trial subjects, transaminitis due to elevation of the transaminases ALT and AST in plasma was observed^24^. However, eriodictyol did not increase the serum levels of ALT or AST in a mouse xenograft model, suggesting that this combination may possess a good clinical safety profile. Eriodictyol is one of the most abundant polyphenols in *Citrus limon* (lemons)^25^ and was reported to be an intermediate metabolite of the catechin synthesis pathway in *Camellia sinensis*^26^. A human pharmacokinetic study demonstrated a plasma concentration of approximately 7 μM after consumption of lemon extract^27^, similar to the tested concentration sufficient to potentiate the apoptosis-inducing effect of EGCG *in vivo*. Furthermore, eriodictyol and its analogues potentiated the anti-MM effect of *O*-methylated EGCG, an EGCG derivative with a significantly longer half-life in blood^28^.

Our result shows 67LR Kocked down significantly attenuated the anti-cancer effect of the combination and 67LR mediates anti-cancer effect of EGCG/Eriodictyol in combination (Supplemental Figure 5A,B). Several reports demonstrated that 67LR mediates the effects of EGCG including anti-multiple myeloma effect^13^,^14^,^16^, anti-acute myeloid leukemia effect^15^,^17^, anti-cervical cancer effect^29^, anti-melanoma effects^12^,^30^,^31^,^32^, anti-inflammatory effect^10^,^33^,^34^,^35^, anti-allergy effect^36^, vascular protection effect^37^ and myoprotectitive effect^38^. Indeed, our preclinical study demonstrated there are significant correlation between the expression level of 67LR and EGCG sensitivity^39^. These reports suggested eriodictyol may potentiate the other effects of EGCG and further investigation is required.

To confirm the effect of the combination in lower level 67LR cells, we evaluated the effect of EGCG/eriodictyol in combination on lower level 67LR cells including normal PBMCs and human malignant pleural mesothelioma cell line ACC-MESO4. Our results demonstrated EGCG/eriodictyol in combination did not show any cytotoxic effect in both lower level 67LR cells (Supplemental Figure 5C–E).

Fractionation approaches such as liquid–liquid and solid-phase extraction are widely used to screen for bioactive compounds from crude extracts (Supplementary Fig. S6). However, this strategy has several drawbacks, such as the requirement for additional fractionation/purification steps that may result in the loss of low abundance but potentially valuable compounds. The chemometric approach described by us may be used for classification and bioactivity assessment without pre-purification for LC–MS (Supplementary Fig. S6). The metabolomics approach allows for the simultaneous analysis of a broad range of low molecular weight components in crude extracts at different concentrations. Combined metabolomic and chemometric studies have been used to characterize the relationships between the metabolomes of crude samples and their attributes, based on the abundance of each metabolite relative to the total abundance of all metabolites^40^,^41^. Theoretically, this methodology enables the identification of multiple metabolites (active compound and positive/negative regulators) that contribute to the extract bioactivity. This approach identified a combination of green tea constituents with potent apoptosis-inducing activity in cancer cells, and may represent a new methodology capable of screening bioactive regulators in crude samples without additional fractionation or purification (Fig. 2, Supplementary Fig. S6).

In summary, we demonstrate an effective strategy for identifying a potentiator of the anti-cancer effect of EGCG in a large number of crude GTEs. Our results show the potential of metabolic profiling and multivariate statistical analyses for evaluating components that contributed to the pharmaceutical effects of GTEs. We identified the EGCG sensitizer eriodictyol that lowers the EGCG IC_50_ within the range of plasma concentrations observed in clinical studies. Thus, this combination of metabolomics and bioassay is a simple and effective methodology that may advance pharmaceutical studies on herbal medicines and botanical drugs.

## Methods

### Study approval

Studies using human tissue samples were approved by the Ethics Committee of the Faculty of Agriculture, Kyushu University. Informed consent from all patients and healthy volunteers was obtained in accordance with the Declaration of Helsinki. All animal studies were conducted in accordance with the law (protocol no. 105) and notification (protocol no. 6) of the Japanese Government for the welfare of experimental animals. All animal experiments were approved by the Animal Care and Use Committee of Kyushu University, Fukuoka, Japan (approval number A24-052-3).

### Chemicals

Propidium iodide (PI), EGCG, superoxide dismutase (SOD) and catalase were obtained from Sigma Aldrich (St. Louis, MO), naringenin from Enzo (Ann Arbor, MI), annexin V-Alexa Fluor 488 from Invitrogen (Carlsbad, CA), theobromine from Wako (Osaka, Japan), eriodictyol cyanidin-3-*O*-galactoside (Cya-gal) and cyanidin-3-*O*-glucoside (Cya-glu) from ExtraSynthese (Riom, France). Hesperetin were provided from Tokyo Chemical Industry (Tokyo, Japan) and epicatechin-3-*O*-gallate (ECG) and epicatechin (EC) were purchased from Mitsuinorin (Tokyo, Japan).

### Preparation of GTEs

The green tea cultivars (Supplementary Table 1) were provided by the National Institute of Vegetable and Tea Sciences, Japan. Dried leaf powder (200 mg) of each green tea cultivar was added to boiling water (10 mL) for 10 min. The extract was filtered through 90-μm filter paper (Advantec, Tokyo, Japan) and centrifuged (1,680 × *g*) for 10 min and was filtered by using a 0.2-μm filter (Sartorius Stedim Biotech, Goettingen, Germany). An extract of the common Japanese green tea Yabukita (YB) was prepared in the same way and used as the control for apoptosis induction tests.

### Cell culture and apoptosis assay

The human MM cell line U266 was maintained in RPMI1640 supplemented with 10% foetal calf serum (Industries, Israel) in a humidified atmosphere with 5% CO_2_ at 37°C. Cells were seeded into 24-well plates (5 × 10^4^ cells/well) and then treated with the indicated extracts or compounds for 96 h in RPMI 1640 medium supplemented with 1% FBS, 200 units/mL catalase and 5 units/mL superoxide dismutase. After 96 h, the relative number of viable cells was estimated using the ATPlite One step^TM^ assay (Perkin–Elmer, Montreal, Canada) according to the manufacturer's instructions. For determination of apoptosis induction, cells were double-stained with annexin V-Alexa Fluor 488 and PI and analysed on a FACS Caliber system (Becton Dickinson, Franklin Lakes, NJ) with FlowJo software (Tree Star, Ashland, OR). The proportion (%) of annexin-V^+^ cells (total apoptotic cells) was calculated by the added annexin V^+^/PI^−^ (early annexin V-positive) and annexin V^+^/PI^+^ (late annexin V-positive) fractions. Protein Kinase B (Akt) kinase activity was measured using a K-LISA Akt Activity Kit (Merck Millipore, Billerica, MA) on an Envision plate reader (Perkin–Elmer). Acidic sphingomyelinase (ASM) activity was measured using BODIPY-C12 (Invitrogen) as described^16^. pLKO.1 vectors encoding scrambled control shRNA or shRNAs targeting 67LR and ASM were purchased from Sigma–Aldrich. Western blotting and knock down assay were performed as previously described^16^.

### Liquid chromatography–mass spectroscopy analysis

GTEs were subjected to LC–MS analysis with an IT-TOF instrument (Shimadzu, Kyoto, Japan) as previously described^42^. Brifely, the equipment was fitted with a Luna C18(2) column (250 mm × 1.0 mm, 5 μm particle size, Phenomenex, Torrance, CA) maintained at 40°C. The mobile phase solvents were 0.05% aqueous formic acid (solvent A) and 0.05% formic acid in methanol (solvent B). Solvent B was increased from 5% to 60% over 7.5 min. Solvent B was then increased from 60% to 100% at 10.1 min. Flow rate was kept constant (0.1 mL/min).

### Multivariate statistical analysis

Mass spectra data obtained by LC–MS were processed using Profiling Solution software (Shimadzu Corporation, Kyoto, Japan) to extract and align peaks. All *m/z* peaks (variables) in either positive or negative ionization mode were separately normalized to the total ion counts (TIC) of each sample. After integration of both TIC-normalized variables, we identified 634 peaks (metabolites) that differed among cultivar samples. The data were mean-centered and scaled to Pareto variance. Datasets of 43 cultivars were subjected to multivariate statistical analysis to identify similarities and differences among samples (e.g. MS datasets). We conducted an unsupervised multivariate principle component analysis (PCA) and a supervised multivariate OPLS analysis were performed with SIMCA-P+ ver.12 (Umetrics, Umea, Sweden). PCA models are shown as score plots and contain two synthetic variables: principal component (PC) 1 and PC2. These plots show groups of samples based on spectral variation.

OPLS regression analysis was performed by using SIMCA-P+. OPLS differs in this respect from PCA, which uses the maximum variation in the metabolite data matrix. The data set was separated into two parts: 36 set samples used to create the model and seven set samples (cultivars 4, 10, 16, 22, 28, 34 and 40), that were not included in the regression model and were used to validate the model's predictive value. The quality of the OPLS model was assessed by the parameter R^2^ and the predictive capacity parameter Q^2^. These values higher than 0.5 indicates good quality. Metabolite peaks were assigned by MS/MS analysis or by searching their masses against online metabolite databases (KEGG, METLIN, or MassBank).

A heat map was generated using Multi-Experiment Viewer (MeV v4.8) (http://www.tm4.org/mev.html). It summarizes the Z-scores of the 634 peaks showing differences among cultivars.

### Xenograft murine model

Seven-week-old female BALB/c mice were obtained from Kyudo (Tosu, Japan). Mice were inoculated subcutaneously in the interscapular area with 1 × 10^6^ MPC-11 mouse myeloma cells. After the appearance of palpable tumours, mice were divided randomly into four groups with an even distribution of tumour sizes and injected i.p. daily with either saline alone, EGCG (15 mg/kg), eriodictyol (15 mg/kg), or with EGCG plus eriodictyol (both at 15 mg/kg) every 2 days. Tumor growth was measured with callipers, and the tumour volume calculated as (length × width^2^)/2.

Aminotransferase (AST) and alanine transaminase (ALT) activities were measured using the transaminase CII-test (Wako Pure Chemical Industries Ltd., Osaka, Japan) to assess heptatoxicity. Immunohistochemical staining and analysis were performed as previously described^16^.

### Statistics

All values are expressed as means ± SEM. Values are the mean of at least three separate experiments in each group. Group means were compared by one-way ANOVA followed by Tukey's test for pair-wise comparisons. A value of *P* < 0.05 was considered statistically significant. IC_50_ was calculated using Calcusyn 2.0 software (Biosoft, Cambridge, UK).

## Author Contributions

M.K., Y.F., S.H., Y.K., K.M., M.T., Y.H., S.Y., M.M. and D.M. performed the experiments, analyzed the data and M.K., Y.F., H.W., M.M., M-Y.M. and H.T. conducted the research. M.K., Y.F. and H.T. wrote the paper. H.T. had primary responsibility for the final content. All authors have reviewed the manuscript.

## Supplementary Material

Supplementary Information Supplementary information
