# Supplementary material for: Metabolic Profiling-based Data-mining for an Effective Chemical Combination to Induce Apoptosis of Cancer Cells
Source: Sci Rep. 2015 Mar 31;5:9474. doi: 10.1038/srep09474 (PMC4379465; doi:10.1038/srep09474)
Supplement: Supplementary Information [file srep09474-s1.pdf]

Metabolic Profiling-based Data-mining for an Effective Chemical Combination to Induce Apoptosis of Cancer Cells

Motofumi Kumazoe[1], Yoshinori Fujimura[2], Shiori Hidaka[1], Yoonhee Kim[1], Kanako Murayama[1], Mika Takai[1], Yuhui Huang[1], Shuya Yamashita[1], Motoki Murata[1], Daisuke Miura[2], Hiroyuki Wariishi[2], Mari Maeda-Yamamoto[3] & Hirofumi Tachibana[1, 2, 4,*]

[1]Division of Applied Biological Chemistry, Department of Bioscience and Biotechnology, Faculty of Agriculture, Kyushu University, 6-10-1 Hakozaki, Higashi-ku, Fukuoka 812-8581, Japan;

[2]Innovation Center for Medical Redox Navigation, Kyushu University, 3-1-1 Maidashi, Higashi-ku, Fukuoka 812-8582, Japan;

[3]National Food Research Institute, National Agriculture and Food Research Organization, 2-1-12 Kannondai, Tsukuba, Ibaraki 305-8642, Japan;

[4]Food Functional Design Research Center, Kyushu University, 6-10-1 Hakozaki, Higashi-ku, Fukuoka 812-8581, Japan

*Corresponding author

Original No.	Cultivar	Apoptosis-inducing activity (%)	Rank	Total Polyphenol (mg/mL)
1	Seishin-oolong	14.6	12	2.33
2	Fukumidori	13.5	14	1.51
3	Benifuji	18.7	8	2.00
4	Minekaori	9.7	29	1.53
5	Benihikari	15.9	10	2.22
6	Minamikaori	10.4	26	1.66
7	Benihomare	23.1	5	2.60
8	Izumi	11.9	19	1.82
9	Fuusyun	11.3	22	1.58
10	Tamamidori	19.4	7	1.59
11	Ohba-oolong	20.0	6	1.81
12	Seishintaipan	15.1	11	3.34
13	Kuritawase	12.4	16	1.88
14	Syunmei	8.5	37	2.59
15	Sayamamidori	13.8	13	2.03
16	Asagiri	10.0	28	1.82
17	Hokumei	10.2	27	1.79
18	Asahi	10.4	25	1.34
19	Sayamakaori	13.5	15	1.79
20	Meiryoku	9.3	30	1.54
21	Kanayamidori	8.8	36	2.09
22	Yamatomidori	8.9	32	1.63
23	Asatsuyu	7.9	42	1.82
24	Toyoka	11.5	21	2.34
25	Yaeho	11.3	23	1.79
26	Ujihikari	8.3	39	1.25
27	Ooiwase	8.3	40	1.29
28	Gokou	8.9	33	1.94
29	Inzatsu131	16.1	9	1.41
30	Surugawase	11.8	20	1.23
31	Samidori	9.1	31	1.71
32	Komakage	11.9	18	1.55
33	Hatsumomiji	25.2	4	1.54
34	Ryoufuu	12.0	17	1.58
35	Minamisayaka	8.3	41	2.38
36	Saemidori	7.6	43	1.37
37	Okuyutaka	8.3	38	1.54
38	Okumidori	7.0	44	1.51
39	Yutakamidori	10.8	24	1.44
40	Yabukita	8.8	34	1.50
41	Benifuuki	29.3	3	1.81
42	Nou6	55.3	1	1.42
43	Sunrouge	50.1	2	1.90

Supplementary Table 1

Rank order of apoptosis induction potency of the leaf extracts from 43 Japanese green tea cultivars on human MM cells.

Apoptosis-inducing effects of GTEs were measured by annexin/PI double staining as described in Figure 1. The percentages of annexin-V^+ cells were calculated by combining annexin V^+/PI^- (early annexin V-positive) and annexin V^+/PI^+ (late annexin V-positive). Apoptotic cells were Original No shows the sample No in Figure1.

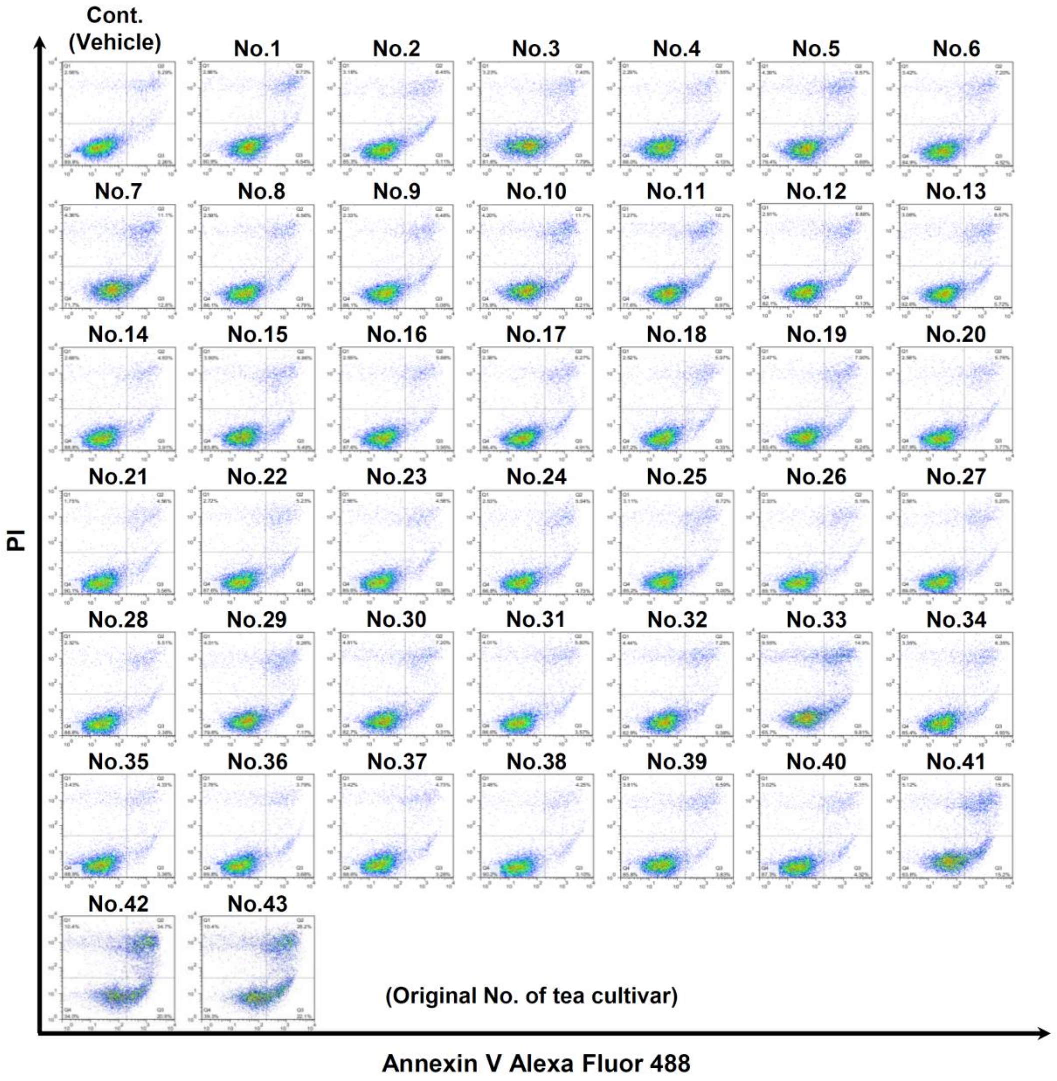

Supplementary Figure 1

Apoptosis induction potency of the leaf extracts from 43 Japanese green tea cultivars on human MM cells.

Apoptosis-inducing effects of GTEs were measured by annexin/PI double staining as described in Figure 1. The percentages of annexin-V^{+} cells were calculated by combining annexin V^{+}/PI^{-} (early annexin V-positive) and annexin V^{+}/PI^{+} (late annexin V-positive). Apoptotic cells were Original No shows the sample No in Figure1.

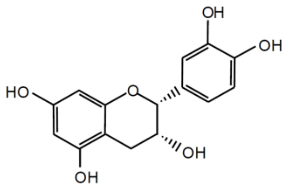

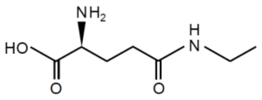

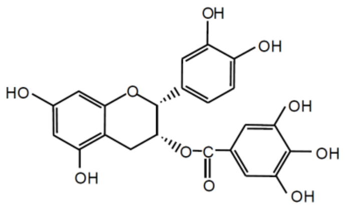

(-)-Epicatechin (EC) **Theanin** **(-)-Epicatechin-3-*O*-gallate (ECG)**

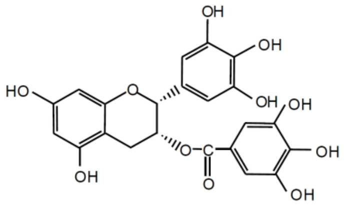

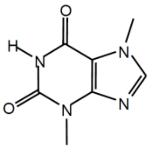

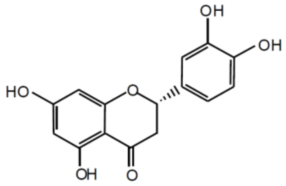

(-)-Epigallocatechin-3-*O*-gallate (EGCG) **Theobromine** **Eriodictyol**

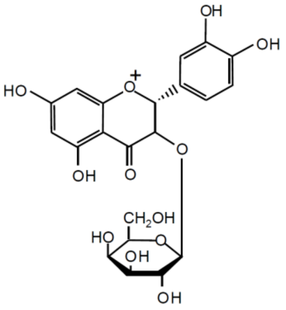

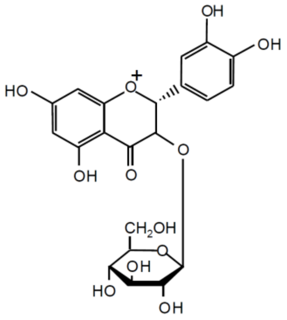

Cyanidin-3-*O*-galactoside (Cya-gal) **Cyanidin-3-*O*-glucoside (Cya-glu)**

Supplementary Figure 2

Chemical structure of metabolites with high VIP values.

Chemical structure of the candidate potentiator of the EGCG anti-MM effect.

A

VIP Rank	VIP Value	Observed *m/z*		Theoretical *m/z*	Compound
1	6.13	289.071	−	289.071 [M − H]⁻	EC
2	5.68	175.106	+	175.108 [M + H]⁺	Theanin
3	5.01	441.083	−	441.082 [M − H]⁻	ECG
4	4.31	345.076	+	—	unknown
5	4.20	392.033	−	—	unknown
6	3.89	479.083	−	—	unknown
7	3.80	459.085	+	459.093 [M + H]⁺	EGCG
8	3.31	337.091	−	—	unknown
9	3.09	457.167	−	—	unknown
10	3.09	457.080	−	457.077 [M − H]⁻	EGCG
11	3.03	337.155	+	—	unknown
12	3.01	181.070	+	181.073 [M + H]⁺	Theobromine
13	2.90	357.102	−	—	unknown
14	2.87	289.066	+	289.071 [M + H]⁺	Eriodictyol
15	2.74	449.102	+	448.100 [M + H]⁺	Cya-gal/-glu

B

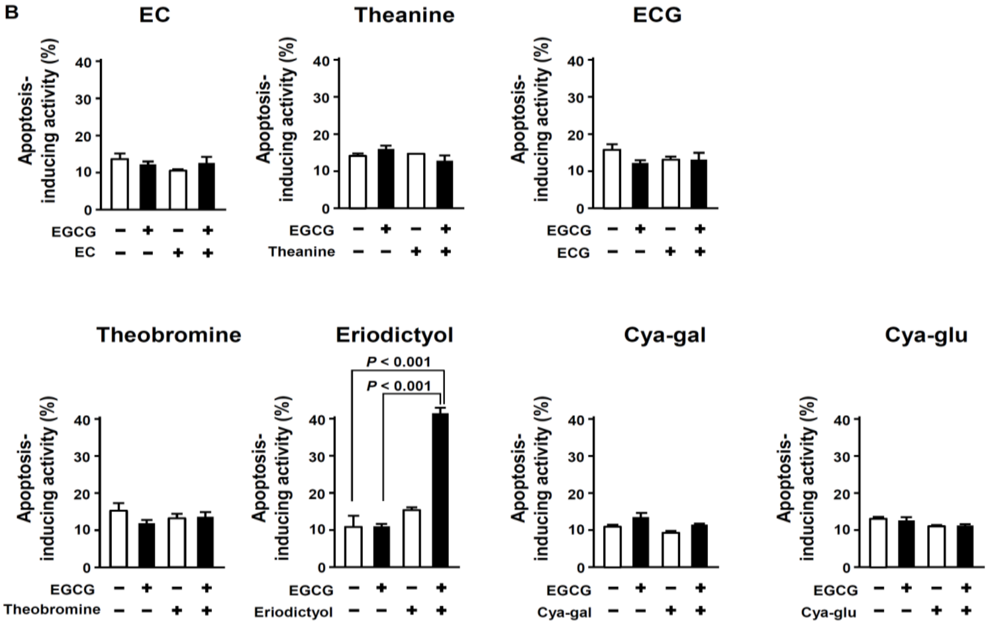

Supplementary Figure 3

The evaluation of the effects of compounds with high VIP values on the anti-MM effect of EGCG.

(A) List of MS peaks focused by successive OPLS regression model. (B) Effect of compounds with high VIP values and EGCG in combination on myeloma cells. Cells were inoculated and then treated with the indicated compounds for 96 h. Cells were then double-stained with annexin V-Alexa Fluor 488 and PI. All data are expressed as mean ± SEM.

A

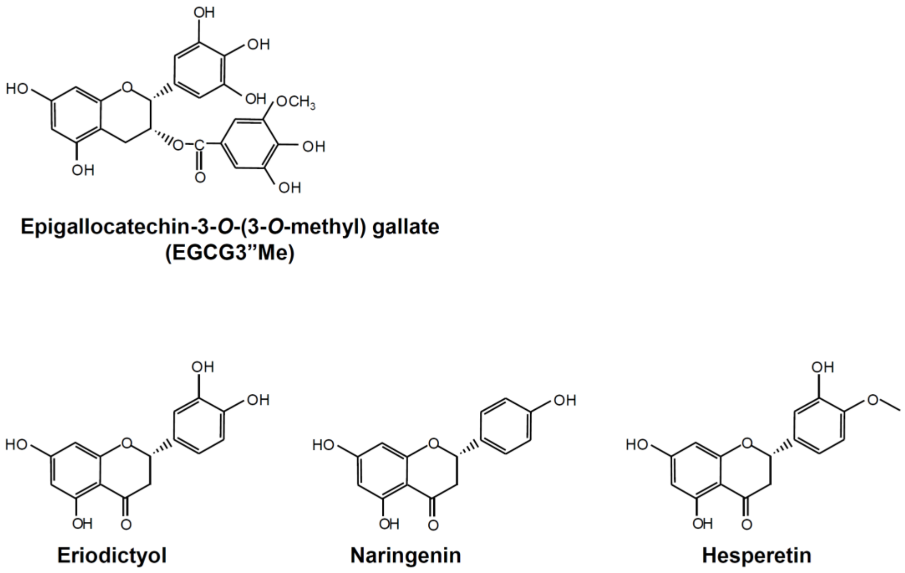

B

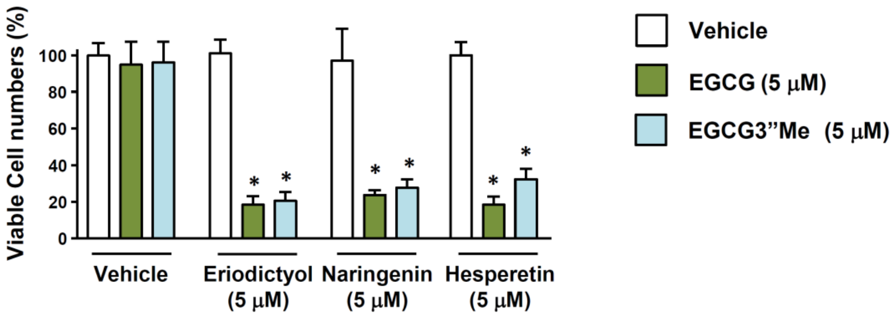

Supplementary Figure 4

Eriodictyol and its analogues potentiate the anti-MM effect of EGCG and its *O*-methylated EGCG analogue.

(A) Structure of the *O*-methylated EGCG derivative and eriodictyol analogues. (B) U266 cells were inoculated into 24-well plates and then treated with the indicated compounds for 96 h. After a 96 h treatment, the ATPlite OneStep assay was performed. All data are expressed as mean ± SEM.

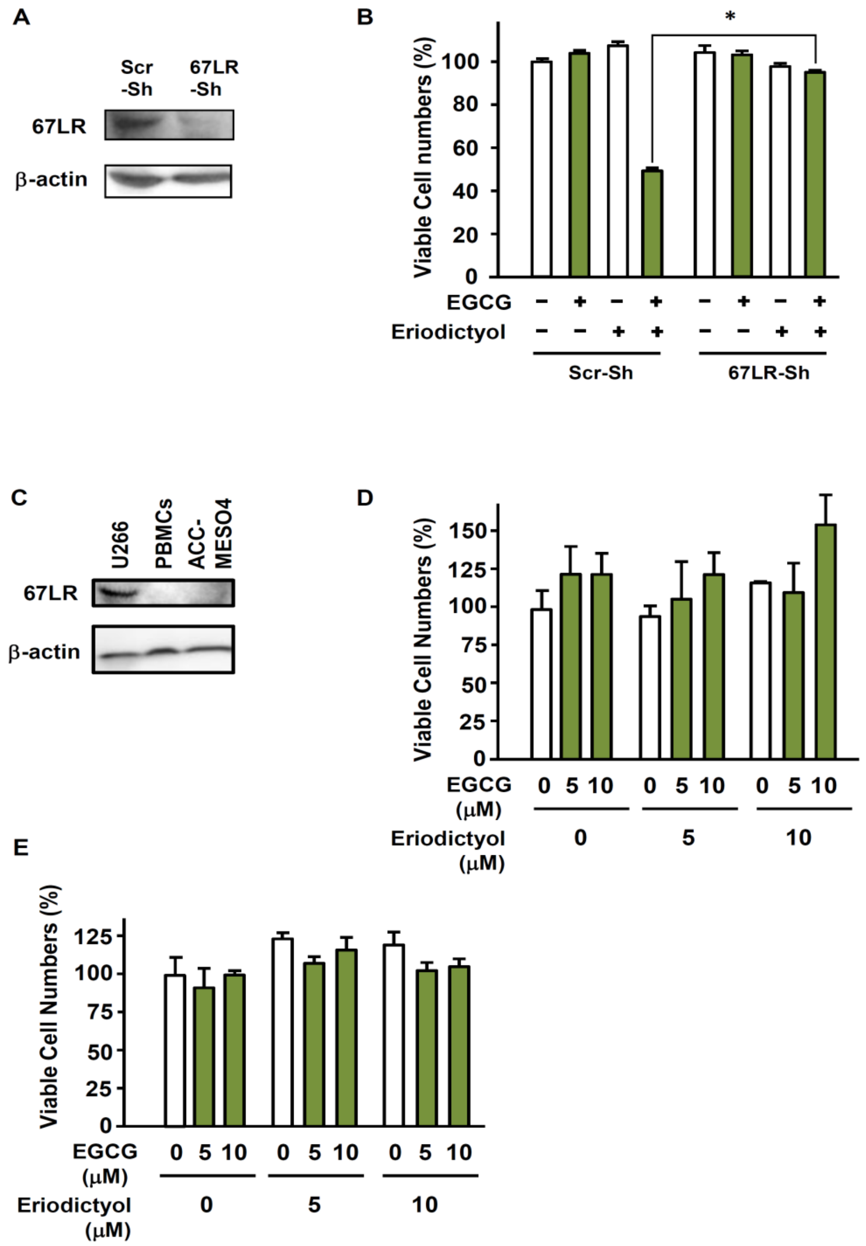

Supplementary Figure 5

67LR mediates anti-cancer effect of EGCG/Eriodictyol in combination.

(A) Immunoblot analyses of 67LR-knockdown in U266 cells. (B) Sensitivity of U266 cells to EGCG (5 μM) and eriodictyol (5 μM) for 96 h after 67LR knock-down. (C) Immunoblot analyses of 67LR. (D) Sensitivity of normal PBMC cells to EGCG and eriodictyol for 96 h. (E) Sensitivity of ACC-MESO4 cells to EGCG and eriodictyol for 96 h.

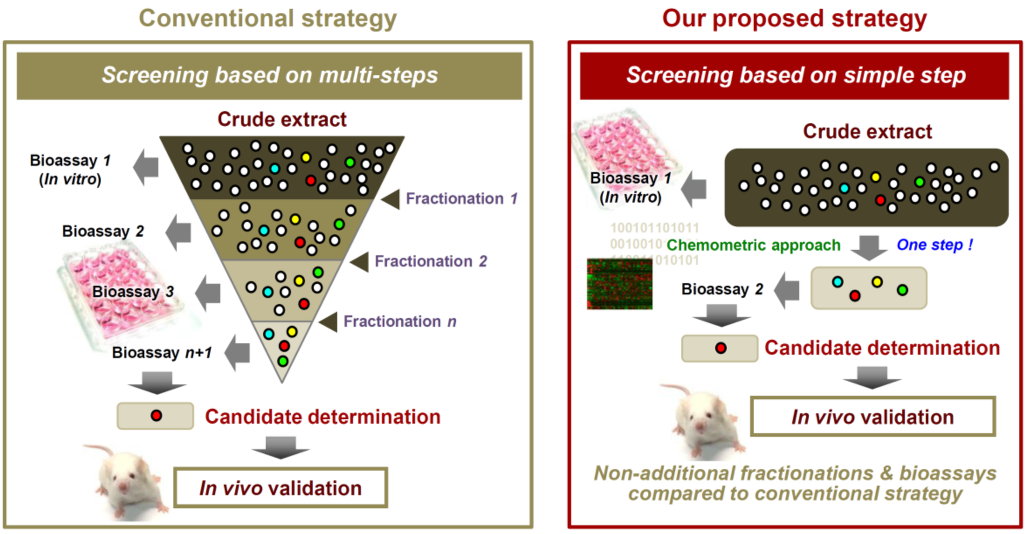

Supplementary Figure 6

Comparison of metabolic profiling-based screening with conventional screening of bioactive compounds. All photographs were taken by Motofumi Kumazoe, Yoshinori Fujimura and Shiori Hidaka.
